# Assessing occupational vaccine uptake and infection control knowledge among healthcare professionals in Cameroon: a cross-sectional analysis

**DOI:** 10.1017/ash.2026.10293

**Published:** 2026-02-25

**Authors:** Syntyche Midrelle Tsague, Leslie Tasha Mbapah, Limunga Aboubakar Khadijatou, Munghieng Tii Ngwachi, Brandon Carl Monika Pouekoua, Sandra Tabe Etaka, Fombo Enjeh Jabbossung, Mbachan Masoeli Takere, Tohson Falake Toh, Aïchatou Menguot Zouleha Salifou, Denis Georges Teuwafeu

**Affiliations:** 1 Faculty of Health Sciences, University of Buea, Buea, Cameroon; 2 Triad Research Foundation (TRF), Buea, Cameroon

## Abstract

**Background:**

Due to occupational exposures, healthcare professionals (HCPs) face an increased risk of infectious diseases, particularly in low-resource settings. Despite infection prevention and control (IPC) policies, systemic and behavioral barriers exist in Cameroon. This study assessed the uptake of occupational vaccines (hepatitis B and COVID-19) and IPC knowledge among HCPs in Fako Division of Cameroon.

**Methods:**

A cross-sectional study was conducted from January to May 2024 among 276 HCPs from four health facilities in Fako Division. Data were collected using a pretested, structured, self-administered questionnaire. Multivariable logistic regressions were employed to identify predictors of good IPC knowledge and combined vaccine uptake. Significance was set at a *P* value of <.05.

**Results:**

Hepatitis B vaccine uptake was 67.4%, while COVID-19 was 32.6%. Doctors had the lowest hepatitis B vaccine uptake (50.7%), while midwives had the lowest COVID-19 vaccine uptake (25.0%), compared with other healthcare cadres. Only 34.8% of HCPs demonstrated good IPC knowledge, despite high reported access to personal protective equipment (PPE) (87.3%) and IPC guidelines (87%). Older age (aOR: 2.41, 95% CI: 1.33–4.39) and previous occupational exposures (aOR: 2.14, 95% CI: 1.17–3.93) were significantly associated with combined vaccine uptake. PPE availability (aOR: 2.64, 95% CI: 1.04–6.74), >7 years of work experience (aOR: 3.22, 95% CI: 1.11–9.35), and contract employment status (aOR: 4.40, 95% CI: 1.47–13.21) were predictors of good IPC knowledge.

**Conclusion:**

The study highlights gaps in occupational vaccine uptake and IPC knowledge among HCPs in Fako, with significant disparities across professional cadres. There is an urgent need for integrated, experience-based IPC training and targeted vaccine advocacy.

## Introduction

Healthcare professionals (HCPs) are among the most vulnerable groups at high risk of exposure to infectious agents due to occupational exposures, making them a priority population for both infection prevention and control (IPC) and immunization programs.^1–3^ A systematic review conducted across 21 African countries found that two-thirds of HCPs had at least one occupational exposure during their career, with both public and private health facilities being equally affected.^3,4^ Effective IPC and vaccination are essential for protecting HCPs, maintaining resilient health systems, and preventing healthcare-associated infections.^5^

Systemic challenges, such as limited IPC infrastructure, irregular supply of personal protective equipment (PPE), and inadequate training on standard precautions, magnify the risk in low- and middle-income countries (LMICs) like Cameroon.^6–9^ Behavioral factors such as high workload and low-risk perception, which reduce adherence to basic precautions, are additional factors to these systemic challenges.^3,8,9^ Along the same line, the burden of vaccine-preventable diseases like hepatitis B and COVID-19 remains significant amongst HCPs, with low vaccination coverage attributed to limited access, vaccine hesitancy, and the absence of institutional policies.^1,7,10^

Although most studies in Cameroon have separately investigated IPC and vaccination regarding healthcare workers’ safety, a significant gap remains in integrated assessments of both IPC knowledge and vaccination status within the same group of HCPs. Understanding the common link between the two domains is essential for designing holistic interventions that address behavioral, structural, and systemic barriers to HCP protection.

This study aimed to assess the uptake of occupational vaccinations (hepatitis B and COVID-19) and IPC knowledge among HCPs in four selected health facilities in the Fako division of Cameroon. The goal was to assist in developing integrated strategies to improve occupational safety and health outcomes among HCPs.

### Materials and method

**Study design**: This cross-sectional study was conducted among HCPs at four selected health facilities in the Fako Division, Cameroon.

**Study period and Populations:** The study was conducted over five months (January to May 2024) among HCPs at four health facilities.

**Study area and setting:** The study was conducted across all units of Buea Regional Hospital (BRH), Limbe Regional Hospital (LRH), Mount Mary Hospital (MMH), and Solidarity Health Foundation (SHF).

Buea Regional Hospital (BRH) is a secondary-level health facility in the South West Region. The hospital has pediatrics, internal medicine, Surgery, Obstetrics, Gynecology, and a laboratory. The HCPs are made up of doctors (30, nurses and midwives (180), laboratory technicians (lab.technician) (30), and pharmacy attendants (06).

The LRH is a secondary health facility and a referral hospital in the SW region. The hospital has a pediatric department, an obstetrics and gynecology department, an internal medicine department, a surgical department, and a laboratory. The HCPs are made up of doctors (39), nurses and midwives (181), lab. technician (36), and pharmacy attendants (06).

Mount Mary Hospital (MMH) is situated in Buea at the foot of Mount Cameroon, and covers. a total catchment area is about 50,000. It has internal medicine, pediatric, maternity, laboratory, and outpatient departments. The HCPs are made up of doctors (05), nurses (26), midwives (13), lab.technicians (09), and pharmacy attendants (02).

Solidarity Health Foundation (SHF) is a private clinic located in Buea. The clinic is made up of medical, surgical, maternity, pediatric, and laboratory. The HCPs are made up of doctors (9), nurses (27), midwives (09), lab. technician (08), pharmacy (05).

### Sampling and sample size determination

After selecting four health facilities via convenience sampling, a minimum sample of 242 was calculated from 607 HCPs using Yamane’s formula. This was increased to 267 to account for a 10% nonresponse rate. A total of 276 HCPs were recruited via simple random sampling across the four previously mentioned health facilities, using a proportionate-to-size sampling approach.

Proportionate to size sampling from each of the four facilities: 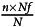


n: minimum sample size of the study population

Nf: total number of healthcare workers in the health facility

N: the total number of healthcare workers in all the hospitals

BRH: 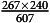 **=**105.56 ∼ 106

LRH: 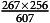 = 112.60 ∼ 113

MMH: 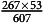 = 23.31 ∼ 24

SHF: 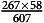 = 25.51 ∼ 26

#### Data collection

A pretested, self-administered, structured questionnaire, designed for this study, was used to collect data on the participants’ sociodemographic characteristics, Vaccination status (hepatitis B and COVID-19), and knowledge of IPC. The assessment of knowledge questionnaire was adapted from a 10-item questionnaire in a study by Mutaru et al., 2022 in Ghana, a similar LMIC context, with a Cronbach’s alpha of .691.^11^

### Study variables

#### Dependent variables

- Proportion of occupational vaccine uptake.
- Knowledge of IPC measures (good, poor).

#### Independent variables

Age, gender (male, female), Cadre (doctor, nurse/midwife, lab.technician), PPE availability (yes, no), previous occupational hazard exposure (yes, no), work experience (<3 yr, 3–7 yr, > 7 yr), training on IPC (yes, no), IPC committee (yes, no), IPC guideline in the facility (yes, no), employment status ( volunteer, contract worker, public worker)

### Study procedure

Two research team members pretested the structured questionnaire among 10 HCPs in a different facility. After obtaining written consent, the structured questionnaire was self-administered to assess participants’ knowledge of IPC practices and occupational vaccination status (hepatitis B and COVID-19).

### Data analysis

Data was cleaned in Excel and exported into StataSE 18.5 for analysis. Categorical variables were presented as frequencies and percentages, and quantitative variables as means with standard deviations or medians with interquartile ranges. A good knowledge was set at or above the mean knowledge score (≥7). Multivariable logistic regression analysis was used to identify factors independently associated with good knowledge and with combined occupational vaccine uptake. Covariates from the univariable model included in the multivariable model for good knowledge had a cut-off *P* < .20. In contrast, combined occupational vaccine uptake was based solely on theoretical relevance to the study. Multicollinearity was assessed using the mean-variance inflation factor: 1.22 for the occupational vaccine uptake model and 1.14 for the good knowledge model. Backward elimination was done to obtain the final full models. The model fitness with Hosmer-Lemeshow goodness of fit for the occupational vaccine uptake model, *P* = .644, and for the good knowledge model, *P* = .246. The level of significance was set at *P* value <.05

### Definition of terms

1. **Occupational vaccine**. These are vaccines required to protect workers, such as HCPs, against diseases related to their jobs. for example hepatitis B, COVID-19, influenza virus. However, this study assessed the uptake of COVID-19 and hepatitis B, which are required in this study setting.
2. **Combined occupational vaccine uptake**. Participants who have received both the COVID-19 and hepatitis B vaccines.
3. **Good IPC knowledge**. Participants with IPC knowledge score of seven or more (based on the mean knowledge score of ≈ 7 in the study).

## Results

In this study, the 18 to 25-year age group was slightly higher at 56.5%. Most participants were female (72.8%), and most workers were volunteers, with 65.6% having worked for 3 to 7 years. Nurses represented 53.3% of the participants, doctors 25.0%, laboratory technicians 13.0%, and midwives 8.7%. In this study, 87.3% of participants had appropriate PPE in their health facilities, 87% had IPC guidelines in their departments, 67.7% had received IPC training, and 69.9% had IPC committees in place. See Table 1.

**Table 1.** General characteristics of participants and facility in Fako division (N = 276)

	Frequency, Percentage	
Variables	*n*	%
Gender		
Male	75	27.2
Female	201	72.8
Age (years)	26.3 ± 6.0	
18 to 25	156	56.5
>25	120	43.5
Professional cadre		
Doctor	69	25.0
Lab. Technician	36	13.0
Midwife	24	8.7
Nurse	147	53.3
Facility		
Buea Regional Hospital	74	26.8
Limbe Regional Hospital	117	42.4
Mount Mary Hospital	58	21.0
Solidarity Hospital	27	9.8
Work status		
Contract worker	66	23.9
State worker	29	10.5
Volunteer	181	65.6
Years of practice		
<3 years	84	30.4
3–7 years	167	60.5
>7 years	25	9.1
Personal protective equipment available		
Yes	241	87.3
No	35	12.7
IPC guidelines present in the department		
Yes	240	87.0
No	36	13.0
IPC training		
Yes	187	67.7
No	89	32.3
IPC committee present in the facility		
Yes	193	69.9
No	83	30.1

### Occupational vaccine uptake among healthcare professionals

In this study, the uptake of the COVID-19 vaccine was 32.6% (95% CI: 27.1–38.5), while the uptake of the hepatitis B vaccine was 67.4% (95% CI: 61.5–72.9). The percentage of HCPs who received at least one vaccine was 78.6% (95% CI: 73.3–83.3). Meanwhile, the proportion of HCPs who received both vaccines was 21.4% (95% CI: 16.7–26.7).

For COVID-19 vaccine uptake, midwives had the lowest proportion (25.0%), followed by doctors (30.4%), with no difference in professional cadre (*P* = .774). More males (36.0%) took the vaccine than females (31.3%) (*P* = .463), and more participants with previous exposure to COVID-19 (41.0%) took the vaccine than those without (29.0%) (*P* = .052). More HCPs with IPC training (34.8%) received the vaccine than those without IPC training (28.1%) (*P* = .269), and participants with more than 7 years of work experience (48.0%) had higher vaccine uptake than their counterparts (*P* = .190). See Table 2.

**Table 2.** Occupational vaccine uptake among healthcare professionals in Fako division

	Vaccine uptake							
	COVID-19		Hepatitis B		≥ One vaccine		Both vaccine	
Variable	%	*P* value	%	*P* value	%	*P* value	%	*P* value
Profession		.774		**.007**		**.002**		.361
Doctors	30.4		50.7		62.3		18.8	
Laboratory technician	36.1		77.8		83.3		30.6	
Midwife	25.0		75.0		87.5		12.5	
Nurse	34.0		71.4		83.7		21.8	
Gender		.463		.190		.190		.516
Female	31.3		69.7		80.6		20.40	
Male	36.0		61.3		73.3		24.00	
Previous exposure		.052		.391		.577		**.008**
Yes	41.0		71.1		80.7		31.3	
No	29.0		65.8		77.7		17.1	
IPC training		.269		**<.001**		**<.001**		.114
Yes	34.8		74.3		85.0		24.1	
No	28.1		52.8		65.2		15.7	
Working experience		.190		**.046**		.084		.099
<3 years	28.6		72.6		77.4		23.8	
3–7 years	32.3		62.3		76.7		18.0	
>7years	48.0		84.0		96.0		36.0	
IPC knowledge		.534		.935		.883		.639
Good	30.2		67.7		78.1		19.8	
Poor	33.9		67.2		78.9		22.2	

%, percentage; IPC, infection prevention and control.

For hepatitis B vaccine uptake, laboratory technicians (77.8%) had the highest proportion, whereas doctors (50.7%) had the lowest (*P* = .007). A higher percentage of participants with IPC training (74.3%) received the vaccine than those without IPC training (52.8%) (*P* < .001). More HCPs with more than seven years of work experience (84.0%) received the vaccine than those with less than three years (72.6%) and those with three to seven years (62.3%) (*P* = .046). See Table 2.

For HCPs who had received at least one vaccine, doctors (62.3%) exhibited the lowest uptake, while midwives (87.5%) showed the highest (*P* = .002). A higher percentage of participants with IPC training (85.0%) received the vaccine than those without IPC training (65.2%) (*P* < .001). More HCPs with previous occupational exposure (31.3%) received both vaccines than those without reported exposure (17.1%) (*P* = .008). See Table 2.

### Factors associated with complete occupational vaccine uptake of healthcare professionals in Cameroon

After controlling for other factors, age greater than 25 years (aOR: 2.41 [95% CI: 1.33–4.39], *P* = .004) and previous accidental exposure (aOR: 2.14 [95% CI: 1.17–3.93], *P* = .014) were independently associated with HCP uptake of complete vaccination (both hepatitis B and COVID-19 vaccine). Good knowledge of IPC (aOR: 0.91 [95% CI: 0.47–1.78], *P* = .789), having trained in IPC (aOR: 1.19 [95% CI: 0.55–2.60], *P* = .657), and working in a facility with an IPC committee (aOR: 1.25 [95% CI: 0.60–2.62], *P* = .548) where not associated with complete occupational vaccination of HCPs. See Table 3.

**Table 3.** Factors associated with complete occupational vaccine uptake of healthcare professionals

	Multivariable analysis (N = 276)			
Variables	%	aOR	(95%CI)	*P*-value
Age (in years)				
18–25	56.5	1		
>25	43.5	2.41	(1.33–4.39)	.004
Gender				
Male	27.2	1.11	(0.54–2.29)	.769
Female	72.8	1		
Profession				
Nurse	53.3	2.49	(0.68–9.18)	.170
Doctor	25.0	2.33	(0.57–9.53)	.237
Laboratory technician	13.0	4.01	(0.95–16.96)	.059
Midwife	8.7	1		
Employment status				
Volunteer	65.6	0.65	(0.24–1.73)	.388
Contract worker	23.9	0.77	(0.28–2.10)	.608
State worker	10.5	1		
Experience (Years)				
<3	30.4	1		
3–7	60.5	0.91	(0.45–1.86)	.797
>7	9.1	1.19	(0.40–3.53)	.757
IPC training				
Yes	67.8	1.19	(0.55–2.60)	.657
No	32.3	1		
IPC committee				
Yes	69.9	1.25	(0.60–2.62)	.548
No	30.1	1		
IPC knowledge				
Good	34.8	0.91	(0.47–1.78)	.789
Poor	65.2	1		
Occupational hazard exposure				
Yes	30.1	2.14	(1.17–3.93)	**.014**
No	69.9	1		

IPC, infection prevention and control; aOR, adjusted odds ratio; %, percentage; N, sample; CI, confidence interval.

### Knowledge of infection prevention and control among healthcare professionals

In this study, the mean IPC knowledge score was 7.05 (SD ±1.12). HCPs with a good knowledge of IPC constituted 34.8% (95% CI: 29.2–40.7).

Laboratory technicians (47.2%) had the highest proportion of good knowledge of IPC, followed by midwives (41.7%). Doctors (27.5%) demonstrated the least knowledge of IPC (*P* = .204). Contract workers were more knowledgeable about IPC (50.0%), whereas state workers were the least knowledgeable about IPC (24.1%) (*P* = .009). Participants with prior occupational hazard exposure were less knowledgeable about IPC (28.9%) than those without previous exposure (37.3%) (*P* = .180). Healthcare professionals (HCPs) in facilities with PPE were more knowledgeable about IPC (37.3%) than those without PPE (17.1%) (*P* = .019). Additionally, HCPs who received IPC training (36.4%) had better IPC knowledge than those without IPC training (31.5%) (*P* = .424). The proportion of participants with IPC guidelines who had good knowledge of IPC was 35.4%, whereas those without IPC guidelines was 30.6% (*P* = .568). See Table 4.

**Table 4.** Proportions of good knowledge of infection prevention and control among healthcare professionals (n = 276)

		Good IPC knowledge		
	N	%	X^2^	*P* value
Total	276	34.8		
Gender			0.0006	.980
Male	75	34.7		
Female	201	34.8		
Age (in years)			0.0355	.851
18 to ≤ 25	156	35.26		
>25	120	34.17		
Profession			4.5927	.204
Doctor	69	27.5		
Laboratory Technician	36	47.2		
Midwife	24	41.7		
Nurse	147	34.0		
Employment status			9.3647	.009
Contract	66	50.0		
State worker	29	24.1		
Volunteer	181	30.9		
Years of practice			4.2006	.122
<3 years	84	29.8		
3–7 years	167	34.7		
>7 years	25	52.0		
PPE available in the facility			5.4982	.019
Yes	241	37.3		
No	35	17.1		
IPC guideline			0.3261	.568
Yes	240	35.4		
No	36	30.6		
IPC training			0.6390	.424
Yes	187	36.4		
No	89	31.5		
IPC committee			0.0971	.755
Yes	193	34.2		
No	83	36.1		
Previous occupational exposure			1.8011	.180
Yes	83	28.9		
No	193	37.3		

X^2^, Chi square; n, number of participants; %, percentage; IPC, infection prevention and control; PPE, personal protective equipment.

### Assessment of knowledge of infection prevention and control practice

In this study, 56.2% of participants answered that gloves confer complete protection, 89.5% answered that all needles should be recapped after use, and 54.7% answered that the safety box should still be used when three-quarters full. See Table 5.

**Table 5.** Knowledge assessment on IPC components among healthcare professionals (n = 276)

	IPC knowledge assessment	
	n = 276	%
Handwash before and after procedure		
Yes	274	99.3
No	2	0.7
Gloves confers complete protection		
Yes	155	56.2
No	121	43.8
Recap all needle after use		
Yes	247	89.5
No	29	10.5
Alcohol based antiseptic as effective as soap and water		
Yes	204	73.9
No	72	26.1
Glove worn before blood/fluid contact		
Yes	256	92.7
No	20	7.3
Waste separation at generation		
Yes	234	84.8
No	42	15.2
Route of TB transmission; airborne		
Yes	257	93.1
No	19	6.9
Change glove before next patient		
Yes	247	89.5
No	29	10.5
Can prepare Chlorine solution		
Yes	198	71.7
No	78	28.3
Safety box use at 3/4 full		
Yes	151	54.7
No	125	45.3

### Factors associated with good knowledge of infection prevention and control among healthcare professionals

In the multivariable regression analysis, HCPs working in a department with PPE (aOR: 2.64 [95% CI: 1.04–6.74], *P* = .042), having more than 7 years of work experience (aOR: 3.22 [95% CI: 1.11–9.35], *P* = .031) and being a contract worker (aOR: 4.40 [95% CI: 1.47–13.21], *P* = .008) were independently associated with good knowledge of IPC. It is worth noting that having complete occupational vaccination was not associated with good knowledge of IPC. Previous occupational hazard exposure was not associated with good knowledge of IPC (aOR: 0.63 [95% CI: 0.35–1.14], *P* = .126). See Table 6.

**Table 6.** Factors associated with good knowledge of infection prevention and control among healthcare professionals in Fako division

	Univariable analysis (n = 276)				Multivariable analysis (n = 276)			
Variables	%	OR	(95%CI)	*P*-value	%	aOR	(95%CI)	*P*-value
Age (in years)								
18–25	56.5	1.05	(0.64–1.73)	.851				
>25	43.5	1						
Gender								
Female	72.8	1.01	(0.58–1.76)	.980				
Male	27.2	1						
Profession								
Nurse	53.3	0.72	(0.30–1.74)	.468		0.70	(0.27–1.81)	.464
Doctor	25.0	0.53	(0.20–1.40)	.201		0.75	(0.26–2.17)	0.590
Laboratory technician	13.0	1.25	(0.44–3.55)	.672		1.10	(0.36–3.36)	.867
Midwife	8.7	1						
Employment status								
Volunteer	65.6	1.41	(0.57–3.49)	.460		2.18	(0.73–6.51)	.164
Contract worker	23.9	3.14	(1.18–8.36)	.022		4.40	(1.47–13.21)	.008
State worker	10.5	1						
Experience (Years)								
<3	30.4	1				1		
3–7	60.5	1.26	(0.71–2.21)	.430		1.38	(0.76–2.52)	.289
>7	9.1	2.56	(1.03–6.37)	.044		3.22	(1.11–9.35)	.031
IPC training								
Yes	67.8	1.24	(0.73–2.13)	.424				
No	32.3	1						
IPC committee								
Yes	69.9	0.92	(0.54–1.57)	.755				
No	30.1	1						
PPE available in facility								
Yes	87.3	2.88	(1.15–7.21)	.024		2.64	(1.04–6.74)	.042
No	12.7	1						
IPC guidelines in the facility								
Yes	87.0	1.25	(0.58–2.66)	.569				
No	13.0	1						
Accidental exposure to sharps and body fluids								
Yes	30.1	0.68	(0.39–1.19)	.181		0.63	(0.35–1.14)	.126
No	69.9	1						
Type of facility								
Private funded	28.3	1.83	(1.07–3.13)	.028		1.39	(0.77–2.51)	.275
Public funded	71.7	1						

PPE, personal protective equipment; IPC, infection prevention and control; TBP, transmission-based precautions; OR, odds ratio; aOR, adjusted odds ratio; n, sample; %, percentage; CI, confidence interval.

## Discussion

This study assessed vaccination status for hepatitis B and COVID-19, and knowledge of IPC HCPs in the Fako division of Cameroon. The findings revealed a disparity in vaccine uptake, with significantly lower uptake for the COVID-19 vaccine compared to the hepatitis B vaccine, and identified a knowledge gap in IPC among HCPs in the study area.

Hepatitis B vaccination uptake was higher (67.4%) compared to COVID-19 vaccine uptake (32.6%) among HCPs. In 2018, Ndongo et al reported that fewer than 25% of HCPs received hepatitis B virus (HBV) vaccination,^1^ which contrasts with our findings and may indicate a marked improvement in HBV prevention among HCPs in Cameroon over the years. However, the higher HBV vaccine uptake in this study may be due to HBV vaccination being considered for at least the first dose. In contrast, in the study by Ndongo et al., three complete doses were considered vaccination. This is similar to the findings by Ogoina et al.,2014 in Nigeria, where they reported that 64.5% of HCPs had received at least a dose of HBV vaccine, and 36.2% had three full doses.^12^ Despite this coverage, up to 31.3% to 48.9% of HCPs are susceptible to HBV, necessitating ongoing efforts to improve coverage.^2,10^

The COVID-19 vaccine uptake in this study was lower than the 42.12% reported among HCPs in a systematic review and meta-analysis in Cameroon by Cheuyem et al., 2025.^13^ It is worth noting that both levels of coverage are below the 70% target set by Cameroon and international partners in 2022.^14^ This low uptake is attributed to vaccine hesitancy. A study in Cameroon by Aseneh et al., 2023 reported factors associated with COVID-19 vaccine hesitancy among HCPs, which were a lower perception of the importance of the vaccine on their personal health, little or no trust in the COVID-19 vaccines, greater concerns about vaccine-related adverse effects, and uncertainty about colleagues’ acceptability of the vaccine.^15^ The low COVID-19 vaccine uptake among HCPs necessitates ongoing efforts to educate them on the importance of vaccination to protect themselves and their patients in high-risk settings, such as healthcare settings.

There were disparities in vaccine uptake among different professional groups. Laboratory technicians had the highest hepatitis B vaccine uptake at 77.8%, while doctors had the lowest, at 50.7%. This may be because laboratory technicians work more frequently with sharps, blood samples, and bodily fluids, making them more aware of the health risks. This contrasts with a systematic review and meta-analysis in Africa, which reports that doctors are more likely to be fully vaccinated against HBV than nurses at 52.4%.^16^ This implies that 1 in 2 doctors in this study setting is susceptible to HBV, indicating the need for more stringent measures to increase vaccine uptake.

The factors identified as significant predictors of combined occupational vaccination uptake (HBV and COVID-19) were age over 25 years and previous accidental occupational exposure. These findings align with other authors who demonstrate that age and past exposures serve as strong motivators for vaccine uptake due to heightened risk awareness.^6^ Contrary to expectations, IPC training, presence of IPC committee, and good IPC knowledge were not significantly associated with combined occupational vaccine uptake. This disconnect between knowledge and practice could be due to systemic barriers, inconsistent training quality, or inadequate reinforcement of IPC principles during clinical activities.

This study found that only 34.8% of HCPs had a good knowledge of IPC measures despite IPC training (67.7%), the availability of PPE (87.3%), and IPC guidelines (87%). These findings are similar to a study in Ghana by Mutaru et al., 2022 where IPC knowledge was low despite adequate training opportunities.^11^ This suggests that it is not enough to train providers and provide IPC materials; ongoing follow-up and knowledge retention over time are also necessary.

Certain misconceptions about IPC knowledge were reported, where 52.2% of HCPs believed that disposable gloves provided complete protection, and 89.5% reported that needles should be recapped after use. In addition, only 45.3% correctly identified that safety boxes should not be used when three-quarters full, which is lower than the 85.2% reported by Mutaru et al., 2022 in Ghana.^11^ These misconceptions are also noted in other studies in Africa, and are linked to higher occupational risks.^9^

PPE availability and having more than 7 years of work experience were predictors of good IPC knowledge. This demonstrates that hands-on experience and resource availability play a significant role in reinforcing IPC principles. In addition, being a contract worker was associated with good IPC knowledge. This is because contract workers often undergo more frequent orientation or refresher training as part of short-term or project-based employment, and may have concerns about job security and contract renewal, which may motivate greater adherence to institutional IPC protocols. However, IPC training was not a predictor of good knowledge. This is in line with findings from the WHO Global Report on IPC, which emphasized the importance of practical, experience-based training over theoretical instructions^8^ as IPC training in this setting is often a one-off session, with insufficient emphasis on practical application and continuous reinforcement.

### Recommendations

1. Stakeholders should enhance IPC training by emphasizing practical application over theoretical knowledge and by conducting regular workshops with hands-on simulations to address misconceptions about PPE use and sharps handling.
2. Focused risk communication strategies to address vaccine hesitancy, most especially among doctors, to improve occupational vaccine uptake.

### Study strength and limitation

The study offers a comprehensive perspective on occupational health readiness by evaluating vaccine uptake (hepatitis B and COVID-19) alongside IPC knowledge.

Study limitations include relying on individuals’ self-reported vaccination status and infection prevention control (IPC) practices, which can introduce social desirability or recall bias and compromise the accuracy of the results. The study was conducted in four conveniently selected health facilities, which limits the generalizability of the findings to all healthcare workers in Fako Division. In addition, the cross-sectional nature of the study precludes establishing causal relationships between the identified factors and IPC knowledge or vaccination uptake.

## Data Availability

The data that supports the findings of this study are available on reasonable request from the corresponding author.
